# Myocardial Work by Speckle Tracking Echocardiography Accurately Assesses Left Ventricular Function of Coronary Artery Disease Patients

**DOI:** 10.3389/fcvm.2021.727389

**Published:** 2021-10-05

**Authors:** Huolan Zhu, Ying Guo, Xiang Wang, Chenguang Yang, Yi Li, Xuyang Meng, Zuowei Pei, Ruisheng Zhang, You Zhong, Fang Wang

**Affiliations:** ^1^Department of Gerontology, Shaanxi Provincial People's Hospital, Shaanxi Provincial Clinical Research Center for Geriatric Medicine, Xi'an, China; ^2^Department of Cardiology, Beijing Hospital, National Center of Gerontology, Institute of Geriatric Medicine, Chinese Academy of Medical Sciences, Beijing, China; ^3^Graduate School, Medical College of Peking University, Beijing, China

**Keywords:** myocardial work, speckle tracking echocardiography, global longitudinal strain, coronary artery disease, heart failure, left ventricular function

## Abstract

**Objective:** Myocardial work (MW) is a novel non-invasive method that uses speckle tracking echocardiography (STE) to assess left ventricular (LV) function. MW incorporates the global longitudinal strain and afterload conditions. Here we aimed to use MW to assess the LV function of patients with coronary artery disease (CAD) with or without heart failure (HF).

**Methods:** We enrolled a total of 150 individuals (50 each) with CAD and a normal LV ejection fraction (LVEF), CAD with HF, and healthy controls. Patients were divided into the hypertension (HTN) and normal blood pressure (no HTN) subgroups. MW was determined from the pressure-strain loop using STE. The relationships between MW indices and conventional echocardiographic parameters were evaluated, and the MW indices were compared among groups.

**Results:** Univariate and multivariate analyses showed that MW indices were strongly correlated with LVEF. The global work index (GWI) was increased in the CAD with normal LVEF subgroup with HTN vs. controls (1,922.3 ± 393.1 vs. 1,639.7 ± 204.6 mmHg%, *p* < 0.05) and decreased in CAD patients with HF (no HTN: 940.9 ± 380.6 vs. 1,639.7 ± 204.6 mmHg%, *p* < 0.05; HTN: 857.3 ± 369.3 vs. 1,639.7 ± 204.6 mmHg%, *p* < 0.05). Global waste work was increased in all CAD subgroups vs. controls. Global constructive work had the same tendency as GWI in patients with CAD. Global MW efficiency was decreased in all patients with CAD.

**Conclusion:** MW using STE accurately quantifies LV function in patients with CAD. It offers additional information about LV function with respect to disease progression, particularly in CAD patients with a normal LVEF.

## Introduction

An accurate assessment of left ventricular (LV) function is crucial in clinical decision-making regarding cardiovascular diseases. This remains a major challenge during disease development. In addition to echocardiography, cardiac magnetic resonance imaging (MRI) and single photon emission computed tomography (SPECT) are used to assess heart function. However, MRI and SPECT are severely limited by their high operational cost, need for expensive and high-maintenance equipment, and radioactivity. In addition to these advantages, echocardiography can provide diagnostic results faster than MRI and SPECT. The conventional echocardiographic parameter, LV ejection fraction (LVEF), is currently recognized as the standard assessment of LV function. However, LVEF is based on movement of the endocardial border, which is less sensitive in the early stage of ischemia. Tissue Doppler imaging (TDI) was used to evaluate heart function before speckle tracking echocardiography (STE) emerged. However, TDI has the disadvantage of its angle dependence leading to inaccurate results. Global longitudinal strain (GLS), which is derived from speckle tracking imaging, is a more sensitive measure of myocardial impairment and ischemia-induced LV function damage and has gradually been used in clinical practice (1). However, GLS may be inadequately interpreted if the LV afterload is increased (2). Myocardial work (MW), which considers the GLS and afterload conditions, offsets the disadvantages of GLS alone for detecting LV dysfunction (3, 4).

Recent studies reported that MW showed distinct patterns in different afterload conditions and settings (4–8). LVEF is usually preserved in the early stages of cardiovascular diseases (9). It is difficult to quantify LV myocardial functional impairments in coronary artery disease (CAD) patients with a normal LVEF. According to Edwards et al., MW can identify early abnormalities in LV function, making it a sensitive index for assessing early LV dysfunction (10). No studies to date have investigated the MW patterns in CAD patients with a normal LVEF and reduced LVEF. The current study aimed to investigate MW indices under different afterload conditions and MW patterns in healthy individuals and CAD patients with different heart functions.

## Materials and Methods

### Study Population

This study enrolled all individuals treated at Beijing Hospital between September 2018 and December 2019. The study protocol complied with the guidelines of the Declaration of Helsinki. All participants provided written informed consent. This study was approved by the Beijing Hospital Ethics Committee. The participants were divided into three groups of 50 each: controls, CAD with normal LVEF, and CAD with heart failure (HF). The control group included healthy individuals from the healthcare management center who had no cardiopulmonary symptoms, had normal electrocardiography findings, and received no medication. Patients with symptoms and diagnosed as HF were assigned to the HF group. The HF group included patients with a mid-range or reduced ejection fraction (11). The inclusion criteria for CAD patients were (1) myocardial ischemia-related symptoms (such as chest pain, chest tightness, palpitation, and shortness of breath); (2) age ≥18 years; and (3) sinus rhythm. The exclusion criteria for CAD patients were (1) ST-segment elevation myocardial infarction; (2) other serious heart disease (congenital cardiomyopathy, moderate to severe valvular disease, malignant arrhythmia, hypertrophic cardiomyopathy, dilated cardiomyopathy, etc.); (3) other end-stage diseases (life expectancy <1 year); (4) coronary angiography findings indicative of myocardial bridge without coronary atherosclerosis; and (5) poor-quality echocardiography images. The CAD patients were divided into hypertension (HTN) and no HTN subgroups. Blood pressure was recorded at the time of the echocardiography. An increased afterload was identified when the brachial systolic blood pressure (SBP) was ≥140 mmHg or diastolic blood pressure (DBP) was ≥90 mmHg. Patients were excluded if they had malicious arrhythmia, valvular disease (moderate or severe), congenital heart disease, or poor-quality echocardiographic images.

### Angiography and Conventional Echocardiography

Angiography was performed after echocardiography by an experienced cardiologist. Stenosis ≥50% in at least one major coronary artery or its main branch was identified as CAD. Echocardiography was performed using a Vivid E95 (GE Vingmed Ultrasound, Norway) according to the guidelines of the American Society of Echocardiography (12). All images were stored in RAW format for offline analysis. LV mitral velocities were obtained using pulsed-wave TDI. The diastolic interventricular septal thickness (IVSd), diastolic posterior wall thickness (PWd), and LV diameter diastole (LVDd) were acquired using two-dimensional (2D) imaging. The LV end-diastolic volume (LVEDV) was measured on the apical four-chamber view. LVEF was calculated using Simpson's method. The transmitral E and A wave velocities were measured using pulsed-waved Doppler

### GLS and MW

GLS and MW were measured using 2D STE. The indices were obtained from four-, two-, and three-chamber views at 45–75 frames/s. The offline analysis was performed using an EchoPAC V203 (GE Vingmed Ultrasound). The MW parameters were obtained using a pressure-strain loop (PSL) area module that was constructed using LV pressure values on the vertical axis and strain on the horizontal axis. Based on previous studies, the peak systolic LV pressure was assumed to be equal to the brachial SBP (3, 4).

**Figure 1:**
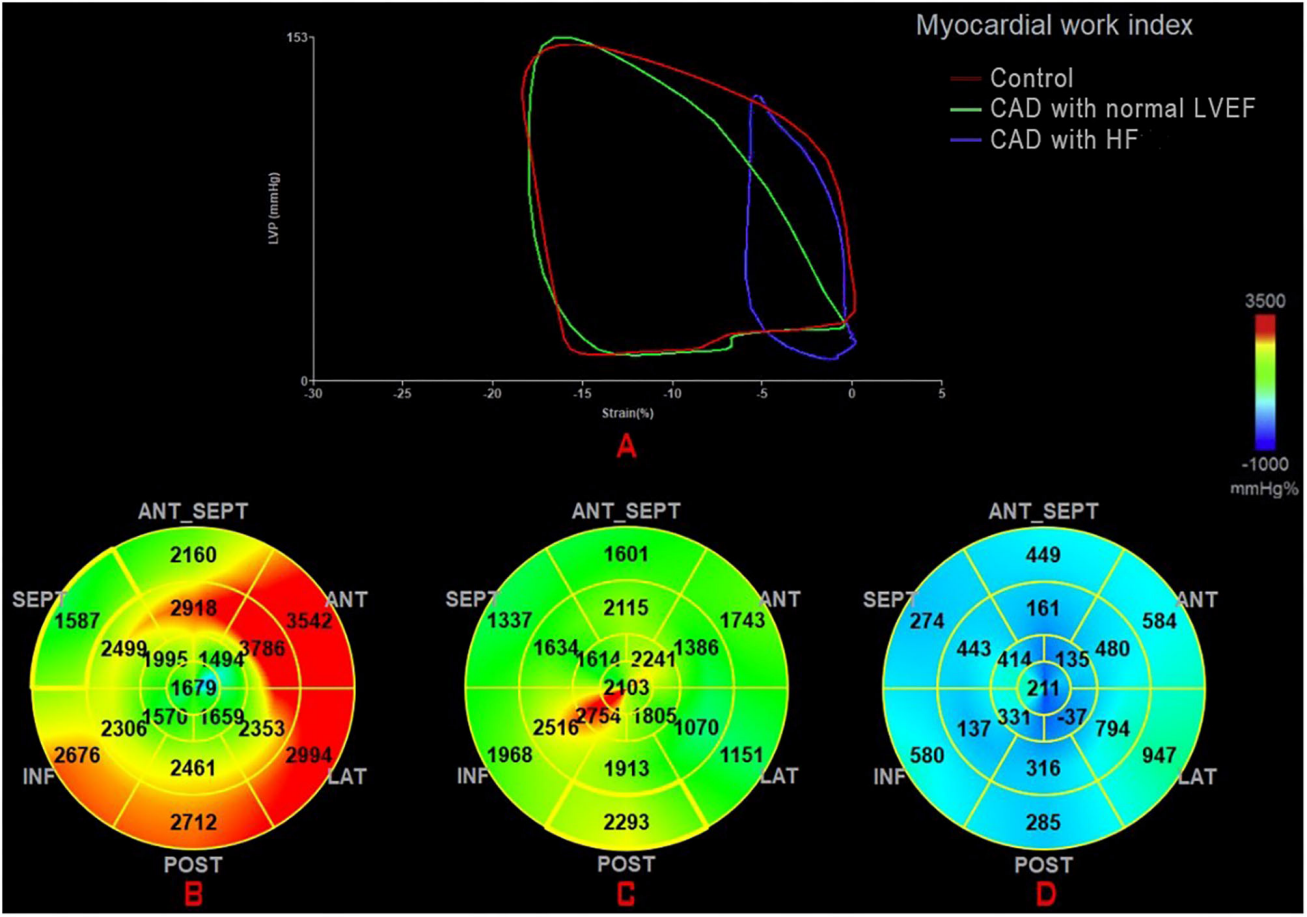
**(A)** Mean PSL in the healthy individual (green) and a CAD patient with normal LVEF (red) and a CAD patient with HF (blue). Seventeen segment bull'seye representation of the MW index (GWI): **(B)** in a healthy individual; **(C)** in a CAD patient with normal LVEF; and **(D)** in a CAD patient with HF. CAD, coronary artery disease; GWI, global work index; HF, heart failure; MW, myocardial work; PSL, pressure-strain loop.

The global work index (GWI) represents the total work within the area of the LV-PSL from mitral valve closure to mitral valve opening (Figure 1). Global constructive work (GCW) represents the work performed during myocardial shortening in systole and lengthening in isovolumic diastole. Global waste work (GWW) represents the work performed by myocytes during myocardial elongation in systole and shortening in isovolumic diastole. Global work efficiency (GWE) is defined as the GCW divided by the sum of the GCW and the GWW: GCW/(GCW + GWW).

### Statistical Analysis

Continuous variables are represented as mean ± standard deviation. The normality of the distribution was tested using the Kolmogorov–Smirnov test. Categorical variables are presented as numbers and percentages. Individual characteristics were compared across subgroups using the χ^2^ test for categorical variables and analysis of variance for continuous variables in relation to the control group. Correlation analysis was performed using Pearson's or Spearman's correlation coefficients. Multivariable linear regression analyses were performed to evaluate the independent correlations between the MW indices and other parameters. Two-sided values of *p* < 0.05 were considered statistically significant. All analyses were performed using SPSS v25.0 (IBM Corp., Armonk, NY, USA).

## Results

### Patient Characteristics and Conventional Echocardiographic Analysis

Patients in the CAD groups were significantly older than those in the control group (*p* < 0.05) (Table 1). The body mass index (BMI) was higher (*p* < 0.05) in the CAD patients with a normal LVEF. The SBP was significantly higher in the HTN subgroup (*p* < 0.05). The DBP was significantly higher in patients in the CAD and normal LVEF with HTN subgroup and lower in patients with CAD and HF without HTN subgroup (*p* < 0.05). The SBP and DBP values differed significantly between the CAD subgroups (*p* < 0.05).

**Table 1:** Patients' demographic characteristics.

	**Control (*n =* 50)**	**CAD with normal LVEF group**		**CAD with HF group**	
		**No HTN (*n =* 26)**	**HTN (*n =* 24)**	**No HTN (*n =* 34)**	**HTN (*n =* 16)**
Age (years)	37 ± 16	63 ± 8.19^*^	64.96 ± 9.10^*^	63.18 ± 9.21^*^	70.04 ± 11.65^*^
BSA (m^2^)	1.73 ± 0.21	1.82 ± 0.15	1.77 ± 0.22	1.79 ± 0.17	1.70 ± 0.18
BMI (kg/m^2^)	23.23 ± 3.35	25.75 ± 2.9^*^	26.65 ± 2.92^*^	24.55 ± 4.51	23.6 ± 3.51
SBP (mmHg)	115.3 ± 11.01	124.77 ± 9.98^*^	150.96 ± 11.03^*^^,^^**^	115.35 ± 13.92	144.63 ± 8.07^*^^,^^**^
DBP (mmHg)	73.78 ± 9.69	70.52 ± 10.01	80.11 ± 10.79^*^^,^^**^	75.23 ± 18.37	67.18 ± 12.99^*^^,^^**^
HR (bpm)	75.52 ± 11.31	73.35 ± 10.68	75.92 ± 11.44	67.97 ± 11.84^*^	81.31 ± 15.7
Male sex	25 (50%)	19 (73.1%)	14 (58.3%)	20 (58.8%)	13 (81.2%)
DM history	—	10 (38.5%)	12 (50.0%)	8 (23.5%)	2 (12.5%)
HTN history	—	5 (19.2%)	22 (91.7%)	21 (61.8%)	7 (43.8%)

* *p < 0.05 vs. the control group*.

** *p < 0.05 in the no HTN vs. HTN group*.

*BMI, body mass index; BSA, body surface area; CAD, coronary artery disease; DBP, diastolic blood pressure; DM, diabetes mellitus; HF, heart failure; HTN, hypertension; LVEF, left ventricular ejection fraction; SBP, systolic blood pressure*.

Patients in the HF subgroup had significantly thicker interventricular septal thickness than the controls (*p* < 0.05) (Table 2). The PWd was higher in HF patients without HTN. The LVDd and LVEDV were significantly increased in patients with CAD and HF. The A wave value was significantly increased (*p* < 0.05) in the CAD subgroup except in HF patients with HTN. The E/A ratio was decreased (*p* < 0.05) in the CAD subgroups except in HF patients with HTN. GLS was significantly impaired (*P* < 0.05) in both HF subgroups. However, no significant differences in GLS were observed in CAD patients with a normal LVEF. The E/e′ was increased (*P* < 0.05) in all patients with CAD.

**Table 2:** Conventional echocardiography parameters.

	**Control (*n =* 50)**	**CAD with normal LVEF group**		**CAD with HF group**	
		**No HTN (*n =* 26)**	**HTN (*n =* 24)**	**No HTN (*n =* 34)**	**HTN (*n =* 16)**
IVSd (mm)	9.18 ± 1.51	9.70 ± 1.02	9.67 ± 1.13	10.77 ± 1.90^*^	10.43 ± 2.21^*^
PWd (mm)	8.86 ± 1.43	9.74 ± 1.25	9.59 ± 1.08	10.00 ± 1.44^*^	9.71 ± 1.42
LVDd (mm)	43.54 ± 4.24	47.48 ± 3.54	45.86 ± 4.13	56.82 ± 8.02^*^	57.39 ± 8.15^*^
LVEDV (mL)	86.62 ± 19.47	105.43 ± 18.11	97.81 ± 21.13	157.63 ± 59.03^*^	166.83 ± 53.75^*^
LVEF (%)	64.14 ± 2.83	62.70 ± 3.64	63.52 ± 3.43	38.14 ± 13.5^*^	36.93 ± 8.86^*^
A wave (cm/s)	0.62 ± 0.14	0.83 ± 0.21^*^	0.84 ± 0.19^*^	0.78 ± 0.26^*^	0.70 ± 0.17
E wave (cm/s)	0.81 ± 0.13	0.73 ± 0.16	0.74 ± 0.16	0.57 ± 0.29^*^	0.76 ± 0.35
E/A ratio	1.38 ± 0.46	0.91 ± 0.77^*^	0.93 ± 0.31^*^	0.92 ± 0.77^*^	1.01 ± 0.51
E/e′	6.59 ± 1.37	10.99 ± 4.50^*^	10.60 ± 5.18^*^	15.00 ± 6.44^*^	15.42 ± 11.00^*^
GLS	−18.26 ± 2.16	−16.78 ± 2.68	−17.25 ± 2.44	−8.09 ± 3.05^*^	−9.04 ± 8.30^*^

* *p < 0.05, significantly different from the control group*.

*GLS, global longitudinal strain; HF, heart failure; HTN, hypertension; IVSd, diastolic interventricular septal thickness; LVDd, left ventricular diameter diastole; LVEDV, left ventricular end-diastolic volume; LVEF, left ventricular ejection fraction; PWd, diastolic posterior wall thickness*.

### Correlations Between MW and Other Parameters

**Figure 2:**
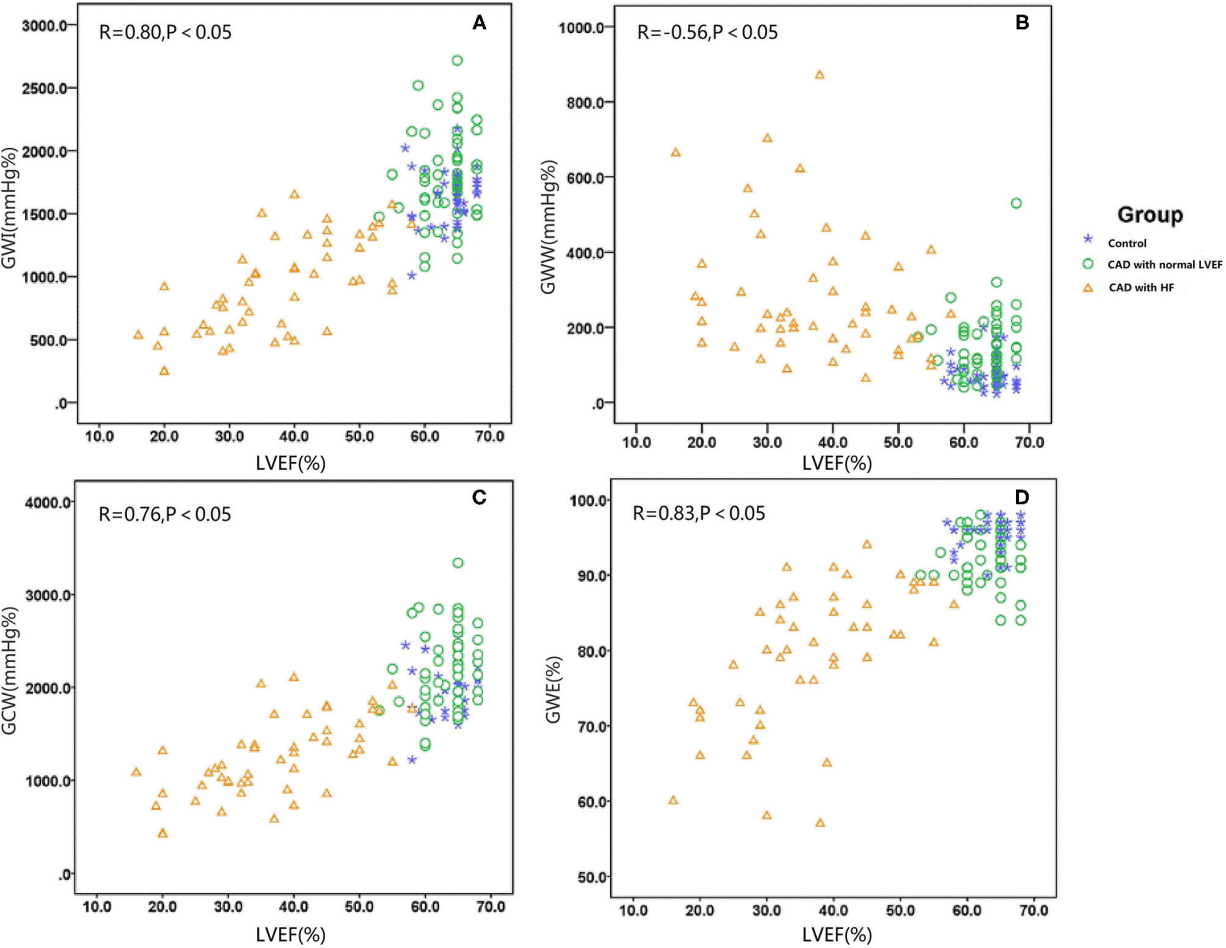
Graph showing the median as well as 25th and 75th percentiles of the GWI **(A)**, GWW **(B)**, GCW **(C)**, and GWW **(D)**. ^*^P < 0.05, significantly different compared with the controls. GCW, global constructive work; GWE, global myocardial work efficiency; GWI, global work index; GWW, global waste work; HF, heart failure; HTN, hypertension.

GWI was strongly correlated with the LVEF and LVDd, moderately correlated with SBP, E wave, and E/e′, and weakly correlated with IVSd (Figure 2A; Table 3). The multivariable analysis revealed that GWI was significantly correlated with LVEF, moderately correlated with SBP, and weakly correlated with DBP and LVDd (Table 3). GWW was strongly correlated with LVEF and LVDd and moderately correlated with age, SBP, IVSd, E/e′, and PWd (Figure 2B; Table 4). GWW was weakly correlated with E/A ratio (Table 4). In the multivariable analysis, GWW was strongly correlated with LVDd and moderately correlated with body surface area and SBP (Table 4). GCW was strongly correlated with LVEF and LVDd, moderately correlated with SBP and E wave, and weakly correlated with E/e′ and IVSd (Figure 2C; Table 5). In the multivariable analysis, GCW was significantly correlated with LVEF and moderately correlated with SBP (Table 5). GWE was strongly correlated with LVEF and LVDd and moderately correlated with age, IVSd, PWd, E wave, E/A ratio, and E/e′ (Figure 2D; Table 6). In the multivariable analysis, GWE was significantly correlated with LVEF and moderately correlated with LVDd. GWE was weakly correlated with body surface area, BMI, SBP, and E/e′ (Table 6).

**Table 3:** Univariable and multivariable analyses of global work index.

**Variable**	**Univariable analysis**		**Multivariable analysis**	
	**Coefficient**	***p*-value**	**Coefficient**	***p*-value**
Sex	0.15	0.066		
Age	−0.149	0.069		
Body surface area	−0.01	0.901		
Body mass index	0.035	0.665		
**SBP**	**0.257**	**0.001**	0.283	<0.001
DBP	0.094	0.251	−0.116	0.025
**IVSd (mm)**	**−0.179**	**0.028**		
PWd (mm)	−0.155	0.059		
**LVDd (mm)**	**−0.674**	**<0.001**	−0.159	0.033
**LVEF (%)**	**0.801**	**<0.001**	0.671	<0.001
A wave (cm/s)	0.097	0.241		
**E wave (cm/s)**	**0.24**	**0.003**		
E/A ratio	0.062	0.447		
**E/e′**	–**0.317**	**<0.001**		

*DBP, diastolic blood pressure; IVSd, diastolic interventricular septal thickness; LVDd, left ventricular diameter diastole; LVEF, left ventricular ejection fraction; PWd, diastolic posterior wall thickness; SBP, systolic blood pressure. p < 0.05 was considered significant*.

**Table 4:** Univariable and multivariable analyses of global waste work.

**Variable**	**Univariable analysis**		**Multivariable analysis**	
	**Coefficient**	***p*-value**	**Coefficient**	***p*-value**
Sex	−0.092	0.264		
**Age**	**0.403**	**<0.001**		
Body surface area	−0.041	0.612	−0.205	0.002
Body mass index	0.086	0.289		
SBP	**0.222**	**0.006**	0.247	<0.001
DBP	−0.018	0.824		
**IVSd (mm)**	**0.24**	**0.003**		
**PWd (mm)**	**0.287**	**<0.001**		
**LVDd (mm)**	**0.594**	**<0.001**	0.625	<0.001
**LVEF (%)**	–**0.556**	**<0.001**		
A wave (cm/s)	0.149	0.069		
E wave (cm/s)	−0.153	0.061		
E/A ratio	−0.186	**0.030**		
E/e′	0.441	**<0.001**		

*DBP, diastolic blood pressure; IVSd, diastolic interventricular septal thickness; LVDd, left ventricular diameter diastole; LVEF, left ventricular ejection fraction; PWd, diastolic posterior wall thickness; SBP, systolic blood pressure. p < 0.05 was considered significant*.

**Table 5:** Univariable and multivariable analyses of global constructive work.

**Variable**	**Univariable analysis**		**Multivariable analysis**	
	**Coefficient**	***p*-value**	**Coefficient**	***p*-value**
Sex	0.133	0.104		
Age	−0.104	0.207		
Body surface area	−0.012	0.882		
Body mass index	0.059	0.466		
**SBP**	**0.315**	**<0.001**	0.345	<0.001
DBP	0.092	0.262	−0.144	0.008
**IVSd (mm)**	–**0.186**	**0.022**		
PWd (mm)	−0.129	0.116		
**LVDd (mm)**	–**0.623**	**<0.001**		
**LVEF (%)**	**0.765**	**<0.001**	**0.761**	**<0.001**
A wave (cm/s)	0.16	0.052		
**E wave (cm/s)**	**0.249**	**0.002**		
E/A ratio	0.034	0.708		
**E/e′**	–**0.254**	**0.003**		

*DBP, diastolic blood pressure; IVSd, diastolic interventricular septal thickness; LVDd, left ventricular diameter diastole; LVEF, left ventricular ejection fraction; PWd, diastolic posterior wall thickness; SBP, systolic blood pressure. p < 0.05 was considered significant*.

**Table 6:** Univariable and multivariable analyses of global work efficiency.

**Variable**	**Univariable analysis**		**Multivariable analysis**	
	**Coefficient**	***p*-value**	**Coefficient**	***p*-value**
Sex	0.112	0.171		
**Age**	–**0.333**	**<0.001**		
Body surface area	0.021	0.793	0.168	0.003
Body mass index	−0.06	0.464	−0.129	0.024
SBP	−0.082	0.321	−0.095	0.026
DBP	0.047	0.568		
**IVSd (mm)**	–**0.234**	**0.004**		
**PWd (mm)**	–**0.248**	**0.002**		
**LVDd (mm)**	–**0.794**	**<0.001**	−0.366	<0.001
**LVEF (%)**	**0.831**	**<0.001**	0.547	<0.001
A wave (cm/s)	−0.111	0.174		
**E wave (cm/s)**	**0.256**	**0.002**		
**E/A ratio**	**0.204**	**0.022**	**−0.233**	**0.014**
**E/e′**	−**0.453**	**<0.001**		

*DBP, diastolic blood pressure; IVSd, diastolic interventricular septal thickness; LVDd, left ventricular diameter diastole; LVEF, left ventricular ejection fraction; PWd, diastolic posterior wall thickness; SBP, systolic blood pressure. p < 0.05 was considered significant*.

### MW by Study Subgroup

**Figure 3:**
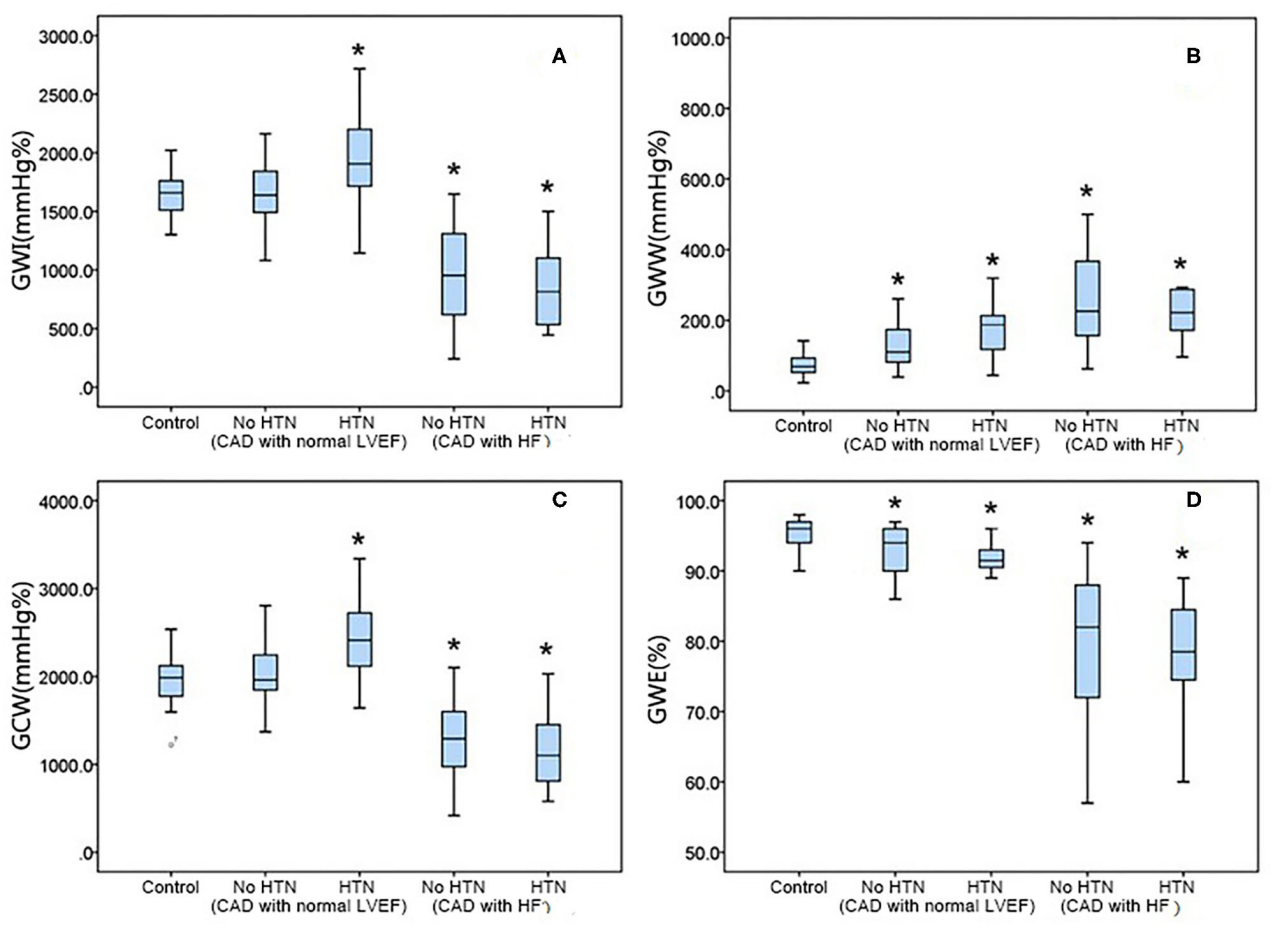
Relationship of the myocardial work indices with the left ventricular ejection fraction. GCW, global constructive work; GWE, global myocardial work efficiency; GWI, global work index; GWW, global waste work; LVEF, left ventricular ejection fraction.

The GWI was significantly elevated in CAD patients with a normal LVEF and HTN compared with controls (HTN: 1,922.3 ± 393.1 vs. 1,639.7 ± 204.6 mmHg%, *p* < 0.05) but not in those without HTN. In the HF group, the GWI was significantly reduced in both subgroups (no HTN: 940.9 ± 380.6 vs. 1,639.7 ± 204.6 mmHg%, *p* < 0.05; HTN: 857.3 ± 369.3 vs. 1,639.7 ± 204.6 mmHg%; Figure 3A).

The GWW was increased in all CAD patients with a normal LVEF vs. controls (no HTN: 124.7 ± 58.1 vs. 79.1 ± 40.3 mmHg%, P0.05; HTN: 183.4 ± 101.8 vs. 79.1 ± 40.3 mmHg%, *p* < 0.05). It also significantly increased in both HF subgroups (no HTN: 274.4 ± 175.9 vs. 79.1 ± 40.3 mmHg%, *p* < 0.05; HTN: 282.6 ± 174.3 vs. 79.1 ± 40.3 mmHg%; *p* < 0.05; Figure 3B).

GCW showed the same tendency as GWI in CAD patients. Compared with the control group, the GCW significantly increased in CAD patients with a normal LVEF and HTN (2,377.5 ± 427.8 vs. 1,964.5 ± 251.3 mmHg%, *p* < 0.05) but not in those with a normal LVEF and no HTN. In the HF group, GCW significantly decreased in all patients (no HTN: 1,275.1 ± 418.8 vs. 1,964.6 ± 251.3 mmHg%, *p* < 0.05; HTN: 1,176.2 ± 423.2 vs. 1,964.5 ± 251.3 mmHg%, *p* < 0.05; Figure 3C).

Compared with the control group, the GWE was decreased in the CAD subgroups (normal LVED but no HTN: 92.9 ± 3.2 vs. 95.2 ± 2.0%, *p* < 0.05; HTN: 91.6 ± 3.3 vs. 95.3 ± 2.0 %, *p* < 0.05) and HF subgroups (no HTN: 80.1 ± 9.7 vs. 95.3 ± 2.0%, *p* < 0.05; HTN: 78.4 ± 8.1 vs. 95.3 ± 2.0%, *p* < 0.05; Figure 3D).

## Discussion

In the current CAD study, MW appeared to be more predictive than LVEF and GLS for assessing LV function. Sub-endocardial fibers are susceptible to ischemia, so repetitive and intermittent minor ischemia may result in subtle forms of myocardial stunning. Minor ischemia may not necessarily result in reduced ventricular wall motion. In such cases, GLS is decreased, while LVEF and regional wall motions are normal (13, 14). As arterial blood pressure rises, the LV must spend more energy to eject the blood. This increase in afterload could reduce the absolute GLS, thus resulting in misinterpretation of the true contraction of the myocardium (2, 15). One study demonstrated that the MW in patients with a high SBP was significantly different from that in controls despite preservation of the strain and EF (4). MW derived from the PSL with consideration of the GLS and afterload enables the accurate assessment of the LV myocardial function.

In our study, the GLS of patients with CAD was lower than that of controls, but the difference was not statistically significant. This lack of a difference may be related to the small sample size. However, MW detected differences between patients with CAD and controls. Furthermore, MW can indicate the subtle and accurate effects of ischemia on the myocardium by combining GLS and blood pressure information. Boe et al. investigated MW in patients with non-ST-segment acute coronary syndromes and reported that a regional GWI <1,700 mmHg% was significantly superior to GLS or LVEF (16). Our results are consistent with this finding in that the MW indices derived from the GLS were more responsive to LV function than GLS.

MW indices were strongly correlated with LVEF in this study. These results are consistent with those of the Normal Reference Ranges for Echocardiography (NORRE) study, which demonstrated strong correlations between GWI and GLS and LVEF (17). In the current study, the GWI was decreased in CAD patients with HF because HF patients are in a decompensated state of heart function due to myocardial damage. The GWI was significantly elevated in patients with HTN because the LV myocardium must compensate for the increased afterload in patients with HTN. Some studies reported that GWI was significantly increased in patients with high blood pressure, consistent with our study results (18, 19). Therefore, GCW can be used to estimate LV performance since it represents the work required for LV ejection. Another study indicated that GCW is significantly correlated with traditional parameters such as LVEF, E/e′, stroke volume, cardiac output, and cardiac index (17). GCW reflects the contractile and viable myocardium and is considered more useful than GLS (20). GWW, as the waste work of myocardium, was significantly elevated in the CAD-HTN subgroup and HF subgroup in our study. In patients with HTN, the increase in GWW may be due to resistance against the increasing afterload. In HF patients, dyssynchronous contractions and post-systolic shortening in the damaged myocardium may contribute to GWW (10). Dyssynchronization in wall motion increases GWW and reduces the ventricular ejection efficiency (21). GWW was reportedly alleviated in dyssynchronous ventricles along with increases in LVEF after cardiac resynchronization therapy (CRT) (3). Another study showed patients with higher GCW exhibit a favorable response to CRT (22). GWE reflects the efficiency of myocardial contraction. In our study, GWE was lower in the CAD subgroups than in controls, particularly in the HF subgroups. The GWI did not differ significantly between the CAD patients with a normal LVEF and HTN and the controls. GWE is considered a better indicator of myocardial impairment than GWI. A reduced GWE results from GCW reductions and GWW increases. GWE can reflect myocardial damage severity and LV function. A previous study reported a strong correlation between LVEF and GWE (8).

MW may provide additional information about dyssynchronous contractions, segmental MW, and myocardial contractility (23). GLS has become an important indicator of heart function and a prognostic factor in CAD patients (24, 25). In the early stages of CAD, MW derived from the combination of GLS and afterload is helpful in the evaluation of myocardial impairment and LV function. In this study, we found a strong correlation between MW and conventional echocardiographic parameters for assessing cardiac function. MW showed a particular pattern in CAD patients. Thus, MW is of great value in evaluating heart function impairments in CAD patients, especially in patients in whom it cannot be identified by conventional echocardiography.

### Limitations

Our study described various patterns of MW in CAD patients with different heart functions. However, the baseline patient ages varied among groups. No study to date demonstrated any effect of age on MW; however, this requires further investigation. Some CAD patients in this study had a history of diabetes and HTN, and it is unclear whether either condition affects MW. Poor image quality limits the assessment and application of MW. In this study, we used only one product (Vivid E95, GE Vingmed Ultrasound). Possible variations in speckle tracking strain findings among products have not been investigated. And finally, this was a small-sample single-center study; thus, larger studies are required to confirm our results.

## Conclusion

MW manifested special patterns in the subgroups of CAD patients with different heart functions under different afterload conditions. Our findings suggest that MW enables an accurate and subtle assessment of ventricular function in CAD patients.

## Data Availability Statement

The data supporting the conclusions of this article will be made available by the authors upon request.

## Ethics Statement

The studies involving human participants were reviewed and approved by Beijing Hospital Ethics Committee. The patients/participants provided their written informed consent to participate in this study.

## Author Contributions

HZ and YG contributed to the study planning and conduct, and the article writing. XW, CY, YL, and XM contributed to the patient enrolment process. ZP and RZ contributed to the data analysis and echocardiography examinations. YZ and FW contributed to the study design and take responsibility for the overall content as guarantors. All authors contributed to the article and approved the submitted version.

## Funding

FW was supported by a grant from the Project of the Ministry of Science and Technology (No. 2020YFC2008106) and the 13th Five-Year National Science and Technology Major Project (2017ZX09304026). YG was supported by a grant from the Beijing Hospital Research Project (No. BJ-2019-133).

## Conflict of Interest

The authors declare that the research was conducted in the absence of any commercial or financial relationships that could be construed as a potential conflict of interest.

## Publisher's Note

All claims expressed in this article are solely those of the authors and do not necessarily represent those of their affiliated organizations, or those of the publisher, the editors and the reviewers. Any product that may be evaluated in this article, or claim that may be made by its manufacturer, is not guaranteed or endorsed by the publisher.
